# HIV Aspartyl Peptidase Inhibitors Interfere with Cellular Proliferation, Ultrastructure and Macrophage Infection of *Leishmania amazonensis*

**DOI:** 10.1371/journal.pone.0004918

**Published:** 2009-03-26

**Authors:** Lívia O. Santos, Fernanda A. Marinho, Ellen F. Altoé, Bianca S. Vitório, Carlos R. Alves, Constança Britto, Maria Cristina M. Motta, Marta H. Branquinha, André L. S. Santos, Claudia M. d'Avila-Levy

**Affiliations:** 1 Laboratório de Biologia Molecular e Doenças Endêmicas, Instituto Oswaldo Cruz (IOC), Fundação Oswaldo Cruz (FIOCRUZ), Rio de Janeiro, Rio de Janeiro, Brazil; 2 Departamento de Microbiologia Geral, Instituto de Microbiologia Prof. Paulo de Góes, Centro de Ciências da Saúde (CCS), Bloco I, Universidade Federal do Rio de Janeiro (UFRJ), Ilha do Fundão, Rio de Janeiro, Brazil; 3 Instituto de Biofísica Carlos Chagas Filho, CCS, (UFRJ), Centro de Ciências da Saúde (CCS), Bloco K, Ilha do Fundão, Rio de Janeiro, Brazil; Columbia University, United States of America

## Abstract

**Background:**

*Leishmania* is the etiologic agent of leishmanisais, a protozoan disease whose pathogenic events are not well understood. Current therapy is suboptimal due to toxicity of the available therapeutic agents and the emergence of drug resistance. Compounding these problems is the increase in the number of cases of *Leishmania*-HIV coinfection, due to the overlap between the AIDS epidemic and leishmaniasis.

**Methodology/Principal Findings:**

In the present report, we have investigated the effect of HIV aspartyl peptidase inhibitors (PIs) on the *Leishmania amazonensis* proliferation, ultrastructure, interaction with macrophage cells and expression of classical peptidases which are directly involved in the *Leishmania* pathogenesis. All the HIV PIs impaired parasite growth in a dose-dependent fashion, especially nelfinavir and lopinavir. HIV PIs treatment caused profound changes in the leishmania ultrastructure as shown by transmission electron microscopy, including cytoplasm shrinking, increase in the number of lipid inclusions and some cells presenting the nucleus closely wrapped by endoplasmic reticulum resembling an autophagic process, as well as chromatin condensation which is suggestive of apoptotic death. The hydrolysis of HIV peptidase substrate by *L. amazonensis* extract was inhibited by pepstatin and HIV PIs, suggesting that an aspartyl peptidase may be the intracellular target of the inhibitors. The treatment with HIV PIs of either the promastigote forms preceding the interaction with macrophage cells or the amastigote forms inside macrophages drastically reduced the association indexes. Despite all these beneficial effects, the HIV PIs induced an increase in the expression of cysteine peptidase b (cpb) and the metallopeptidase gp63, two well-known virulence factors expressed by *Leishmania* spp.

**Conclusions/Significance:**

In the face of leishmaniasis/HIV overlap, it is critical to further comprehend the sophisticated interplays among *Leishmania*, HIV and macrophages. In addition, there are many unresolved questions related to the management of *Leishmania*-HIV-coinfected patients. For instance, the efficacy of therapy aimed at controlling each pathogen in coinfected individuals remains largely undefined. The results presented herein add new in vitro insight into the wide spectrum efficacy of HIV PIs and suggest that additional studies about the synergistic effects of classical antileishmanial compounds and HIV PIs in macrophages coinfected with *Leishmania* and HIV-1 should be performed.

## Introduction

Leishmaniasis is among the most neglected of the tropical diseases: more than 12 million people are currently infected worldwide, there are 2 million new cases every year (a number that is growing), and 350 million people are considered to be at risk. Two basic clinical forms are recognized – cutaneous leishmaniasis (CL), a disfiguring and stigmatizing disease, and visceral leishmaniasis (VL) or kala-azar, which is fatal without treatment [1]. The development of the HIV/AIDS pandemic during the last 20 years has modified the spectrum of leishmaniasis in both the clinical and epidemiological fields [2]. Cutaneous leishmaniasis, for instance, has attracted more attention due to reactivation, or even visceralization, in the immunocompromised hosts [2]. Currently available therapeutic options range from topical treatment to systemic therapy for more complex cases. These agents are expensive, not active orally, require long-term parenteral administration and produce serious side effects (e.g. cardiac and renal toxicity); moreover, resistance to these compounds has become a severe problem [3].

In recent years, the introduction of anti-retroviral drugs, particularly aspartyl peptidase inhibitors (PIs) used in the chemotherapy of HIV, into medical practice has led to a marked improvement in the life expectancy of AIDS sufferers by the fall of HIV viremia and by restoring the immune responses with an increase in the number of CD4^+^ T lymphocytes [4]–[7]. In addition, the functional upgrading of two critical components of innate antimicrobial immunity, such as neutrophils and monocytes, may contribute to the improved cell-mediated immune responses against opportunistic infections in highly active anti-retroviral therapy (HAART)-treated patients [5].

Indeed, after the introduction of PIs in the antiretroviral therapy for HIV, the number of coinfected cases reported in European *Leishmania*-endemic countries fell sharply. Nevertheless, the disease still constitutes an issue in patients who have AIDS, being the third most frequent parasitic opportunistic infection in Europe [8]. The drastic reduction in the incidence, morbidity and mortality of AIDS coinfections after the introduction of PIs in the antiretroviral therapy was the first line of evidence that these compounds could exert a direct effect on opportunistic pathogens [9], which was demonstrated in several fungi, such as *Cryptococcus neoformans* [10], *Candida albicans* [11] and *Fonsecaea pedrosoi* [12], and also in the protozoa *Plasmodium falciparum* [13]. Finally, it was recently demonstrated that two PIs (indinavir and saquinavir) have a direct effect on *Leishmania infantum* and *Leishmania major* promastigotes in vitro, which are responsible for visceral and cutaneous manifestations, respectively [14].

*Leishmania* species are dimorphic protozoa existing as promastigote and amastigote forms, which survive in the lumen of the digestive system of the sand fly and within the phagolysosomal compartment of mammalian macrophages, respectively. In the mammalian host, these protozoa are obligate intracellular parasites of cells from the macrophage-dendritic cell lineages [15]. Since *Leishmania* and HIV infect the same target cells, it is believed that complex interactions between these pathogens take place.

Peptidases participate in several physiological and pathological processes in different cell types. In *Leishmania*, this class of hydrolytic enzymes directly acts in different steps of the microorganism-host interplay, being considered as virulence factors [16]–[18]. Considering all these facts together, we have conducted a study to investigate the direct effect of five different HIV PIs (indinavir, saquinavir, nelfinavir, lopinavir and amprenavir), commonly used in HAART, on *L. amazonensis* growth ability, ultrastructure, interaction of this human pathogen with mouse peritoneal macrophages in vitro, and expression of peptidases, which are classical leishmanial virulence factors.

## Methods

### Chemicals

Saquinavir and nelfinavir were obtained from Hoffmann-La Roche AG (Grenzach-Wyhlen, Germany), indinavir from Merck Sharp & Dohme GmbH (Haar, Germany), lopinavir from Abbott Laboratories (Abbott Park, IL, USA) and amprenavir from GlaxoSmithKline (NC, USA). Anti-α-tubulin monoclonal antibody, bovine serum albumin (BSA), dimethylsulfoxide (DMSO), heat-inactivated fetal bovine serum (FBS), 3-(4,5-dimethylthiazol-2-yl)-2,5-diphenyl tetrazolium bromide dye (MTT), dithiothreitol (DTT), ethylenediaminetetraacetic acid (EDTA), propidium iodide, Schneider's insect medium, 3-[(3-cholamidopropyl)-dimethylammonio]-1-propanesulfonate-Chaps (CHAPS) and HIV-1 peptidase substrate [Arg-Glu(EDANS)-Ser-Gln-Asn-Tyr-Pro-Ile-Val-Gln-Lys(DABCYL)-Arg] were purchased from Sigma Chemical Co. (St Louis, USA). Media constituents, buffer components, reagents used in electrophoresis and immunoblotting were purchased from GE Life Care (Little Chalfont, UK). All other reagents were analytical grade or superior.

### Parasite culture

*Leishmania amazonensis* (MHOM/BR/77/LTB0016 strain) was obtained from *Leishmania* Type Culture Collection (Fundação Oswaldo Cruz, Rio de Janeiro, RJ, Brazil). Promastigote forms were maintained by weekly transfers in 25-cm^2^ culture flasks with Schneider's insect medium, pH 7.0, supplemented with 10% FBS at 26°C.

### Multiplication inhibition assay

The effects of five distinct HIV aspartyl PIs (amprenavir, indinavir, lopinavir, nelfinavir and saquinavir) on promastigote forms of *L. amazonensis* were assessed by a method similar to that described previously [19]. Promastigotes were counted using a haemocytometer and re-suspended in fresh medium to a final concentration of 5×10^7^ viable promastigotes/ml. Viability was assessed by mobility and lack of staining after challenge with trypan blue. Each inhibitor compound was added to the culture at final concentrations ranging from 15 µM to 500 µM (starting from a 20 mM solution in DMSO that was serially diluted in culture medium). Alternatively, promastigotes were also treated with different concentrations (25, 50 and 100 µM) of pepstatin A (a classic aspartyl peptidase inhibitor). Dilutions of DMSO corresponding to those used to prepare the drug solutions were assessed in parallel. After 24, 48, 72 and 96 hours of incubation at 26°C, the number of viable motile promastigotes was quantified by counting the flagellates in a Neubauer chamber. The 50% inhibitory concentration (IC_50_), i.e. the minimum drug concentration that caused a 50% reduction in survival/viability in comparison with that in identical cultures without the compound, was evaluated after 48 hours for the most effective PIs. These values were determined by linear regression analysis by plotting the number of viable promastigotes versus log drug concentration using Origin Pro 7.5 computer software.

### Viability assays

The effect of the HIV PIs on the murine macrophage viability was evaluated by an MTT micromethod described previously [20]. First, peritoneal macrophages from female BALB/c mice (6–8 weeks old) were collected in cold phosphate-buffered saline (PBS; 150 mM NaCl, 20 mM phosphate buffer, pH 7.2) and 5×10^5^ cells were allowed to adhere in 96-well tissue culture plates for 1 hour at 37°C, in a 5% CO_2_ atmosphere. Non-adherent cells were removed by washes with PBS and the wells refilled with DMEM medium supplemented with 10% FBS. Macrophages were then incubated with increasing concentration of PIs (1.56 to 25 µM) in triplicate. After 24 hours, the medium was discharged and the formation of formazan was measured by adding MTT (5 mg/ml in PBS, 50 µg/well) and incubating the wells for 3 hours in the dark at 37°C. The plates were subsequently centrifuged at 1100 rpm for 7 minutes, the supernatant was removed, the pellet was dissolved in 200 µl of DMSO and the absorbance measured in an ELISA reader at 490 nm (Bio-Tek Instruments). Concentrations of the HIV PIs capable of maintaining 95% macrophage viability were used in the interaction assays.

To determinate the promastigotes viability, parasites were resuspended in Schneider's insect medium supplemented with 10% FBS in 96-well plates (1×10^7^ promastigotes/well), and incubated at 26°C with increasing concentrations of HIV PIs (50–250 µM) in triplicate. After 1–4 hours, viable cells were estimated by propidium iodide staining. In this last methodology, cell death due to a loss in cell membrane integrity of drug-treated *L. amazonensis* was assessed by flow cytometry measuring the level of propidium iodide uptake into damaged promastigotes. Concentrations of the HIV PIs capable of maintaining at least 95% of living promastigotes were used in the experiments. Experiments were carried out in accordance with protocols approved by the Institutional Animal Care and Use Committee at Universidade Federal do Rio de Janeiro and Fundação Oswaldo Cruz (CEUA L-0006/07).

### *Leishmania*-macrophage interaction assay

Stationary-phase promastigotes were washed with PBS, counted in a Neubauer chamber, added to the macrophage culture well plates at a parasite to cell ratio of 10∶1, and incubated for 2 hours at 37°C in a 5% CO_2_. The free parasites were removed by successive washes with PBS. Thereafter, *Leishmania*-infected cells were treated for 24 hours with PIs that had a prominent effect on promastigote growth, at final concentrations of 6.25, 12.5 and 25 µM of nelfinavir and 3.125, 6.25 and 12.5 µM of amprenavir and lopinavir (concentrations of the HIV PIs capable of maintaining 95% macrophage viability). After the interaction, cells were washed with PBS at 37°C, the coverslips were fixed in methanol and stained as described below. Alternatively, parasites were pre-treated with PIs for 1 hour with sub-inhibitory drug concentrations (50 and 100 µM of nelfinavir and lopinavir; 100 and 200 µM of amprenavir), washed three times in PBS and then added to the macrophage culture in well plates. After 1 hour of interaction, cells were fixed in methanol, Giemsa-stained and dehydrated in acetone solutions progressively replaced by xylol. The assembly of the slides was done with Permount. The percentage of infected macrophages was determined by randomly counting at least 200 cells in each of duplicated cover slips. Experiments were repeated three times. The association index was obtained by multiplying the percentage of infected macrophages by the number of amastigotes per infected macrophage. Experiments were carried out in accordance with protocols approved by the Institutional Animal Care and Use Committee at Universidade Federal do Rio de Janeiro and Fundação Oswaldo Cruz (CEUA L-0006/07).

### Electron microscopy

Control and HIV PIs-treated parasite cells were cultured as described above and promastigotes were incubated with nelfinavir or lopinavir at the IC_50_ concentration. The possible parasite ultrastructure modifications along different time periods of treatment with HIV PIs were performed. In this context, promastigote cells (1×10^8^) were treated for 2, 4, 6, 8, 12 and 24 hours and then fixed overnight at 4°C in 2.5% glutaraldehyde in 0.1 M cacodylate buffer, pH 7.2. After fixation, cells were washed in cacodylate buffer and postfixed for 1 hour in 0.1 M cacodylate buffer containning 1% osmium tetroxide, 0.8% potassium ferrocyanide and 5 mM CaCl_2_. The cells were then washed in the same buffer, dehydrated in acetone, and embedded in Epon. Ultrathin sections were mounted on 300-mesh grids, stained with uranyl acetate and lead citrate and observed under a Zeiss 900 transmission electron microscope (Zeiss, Oberkochen, Germany).

### Evaluation of peptidase expression by gelatin-SDS-PAGE

Promastigotes (1×10^8^ cells) were incubated with PIs (nelfinavir, lopinavir and amprenavir) for 4 hours at 26°C at a final concentration of 100 µM. The gels were loaded with 5×10^6^ cells, which were added to SDS–PAGE sample buffer (125 mM Tris, pH 6.8, 4% SDS, 20% glycerol and 2% bromophenol blue). Electrophoresis was performed at a constant current of 60 mA at 4°C for 2 hours. After electrophoresis, SDS was removed by incubation with 10 volumes of 1% Triton X-100 for 1 hour. Subsequently, the gels were incubated in 50 mM sodium phosphate buffer, pH 5.5, supplemented with 2 mM DTT at 37°C. After incubation for 48 hours, the gels were washed twice with distilled water, stained for 2 hours with 0.2% Coomassie brilliant blue R-250 in methanol-acetic acid-water (50∶10∶40), and destained overnight in a solution containing methanol-acetic acid-water (5∶10∶85), to intensify the proteolytic halos. The gels were dried, photographed and the density profiles digitally processed [21].

### Evaluation of peptidase expression by immunoblotting

Promastigote forms treated or not with PIs, as described above, were added to SDS–PAGE sample buffer and mixed with 10% β-mercaptoethanol, followed by heating at 100°C for 5 minutes. Thereafter, protein extracts (equivalent to 5×10^6^ cells) were separated in 12% SDS–PAGE and the polypeptides electrophoretically transferred at 4°C at 100 V/300 mA for 2 hours to a nitrocellulose membrane. The membrane was blocked in 5% low-fat dried milk in PBS containing 0.5% Tween 20 (PBS/Tween) for 1 hour at room temperature. Then, membranes were washed three times (10 min each) with the blocking solution and incubated for 2 hours with the following polyclonal antibodies: anti-cpb (1∶1000) raised against *Leishmania mexicana* cysteine peptidase B (kindly provided by Dr Mary Wilson – Department of Internal Medicine, Biochemistry, Microbiology and Epidemiology, Program in Molecular Biology, University of Iowa, USA), anti-gp63 (1∶2000) raised against *L. mexicana* (kindly provided by Dr Peter Overath – Max-Planck-Institut für Biologie, Abteilung Membranbiochemie, Germany), and anti-α-tubulin monoclonal antibody at 1∶500. The secondary antibody used was peroxidase-conjugated goat anti-rabbit IgG at 1∶20000 followed by chemiluminescence immunodetection after reaction with ECL reagents [21]. The relative molecular mass of the reactive polypeptides was calculated by comparison with the mobility of SDS–PAGE standards. The X-ray films were photographed and the density profiles digitally processed [21].

### Aspartyl peptidase assay

The enzymatic activity over HIV-1 peptidase substrate was determined using *L. amazonensis* extract, which was obtained by repeated freeze-thawing cycles of cells in 10 mM Tris-HCl, pH 7.2, containing 1% CHAPS. Then, the cellular extract was incubated for 40 min at 4°C, centrifuged at 10,000 g for 30 min at 4°C, and stored at −70°C in aliquots for no longer than 4 days. Cleavage of HIV-1 peptidase substrate was monitored continuously in a spectrofluorometer (SpectraMax Gemini XPS, Molecular Devices, CA, USA) using an excitation wavelength of 340 nm and an emission wavelength of 490 nm. A 500 µM stock solution of the fluorogenic substrate sample was prepared in DMSO. The reaction was started by the addition of 2 µM substrate to the parasite extract (10 µg) in a total volume of 60 µl of 100 mM sodium acetate, 1 M sodium chloride, 1 mM EDTA, 1 mM DTT, 10% DMSO, 1 mg/ml BSA, pH 4.7, in the presence or absence of nelfinavir, amprenavir, lopinavir or pepstatin A. The reaction mixture was incubated at 37°C for 15 min. The assays were controlled for self-liberation of the fluorophore over the same time interval.

### Statistical analysis

All experiments were repeated at least three times, and representative images of these experiments are shown. The data were analyzed statistically using Student's *t* test using EPI-INFO 6.04 (Database and Statistics Program for Public Health) computer software. *P* values of 0.05 or less were considered statistically significant.

## Results

### Effect of HIV PIs on the growth

**Figure 1:**
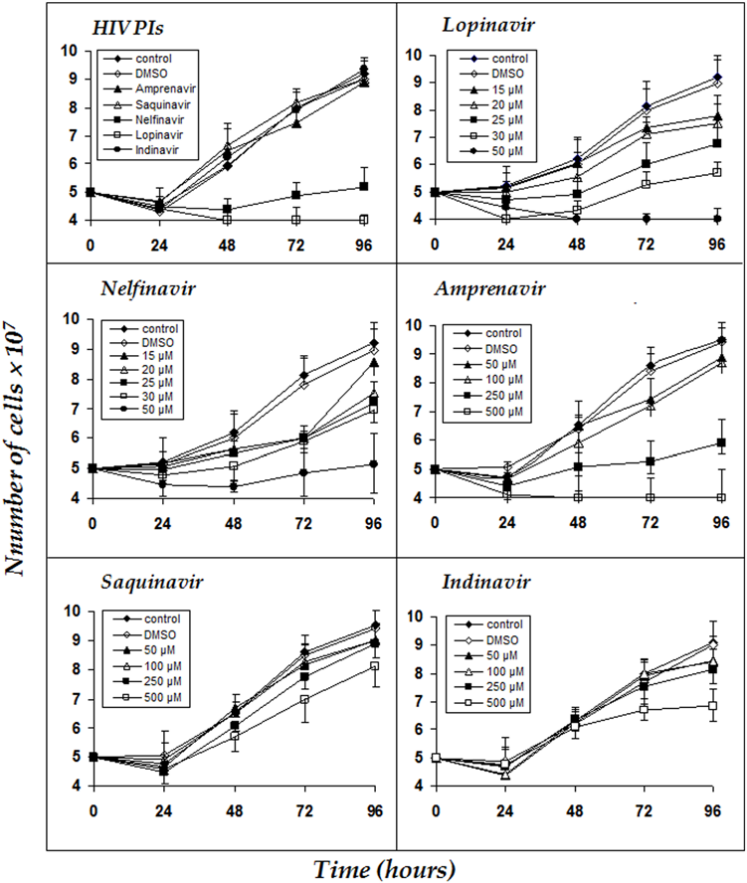
Effect of HIV PIs on the growth rate of *Leishmania amazonensis.* The growth pattern of *L. amazonensis* was followed for parasites cultivated at 26°C in the absence (control) or presence of lopinavir, nelfinavir, amprenavir, saquinavir and indinavir at 50 µM (HIV PIs). Subsequently, lopinavir and nelfinavir were assayed in concentrations ranging from 15 to 50 µM. Amprenavir, saquinavir and indinavir were tested in concentrations ranging from 50 to 500 µM. The PIs were added to the cultures at 0 hour and viable cells were counted daily by trypan blue exclusion and motility. In all experiments, the data from DMSO represents the concentration present in the highest dose of each compound. Data shown are the mean±standard error (S.E.) of three independent experiments performed in triplicate.

In order to establish whether the aspartyl HIV PIs might have any effect on *L. amazonensis* multiplication, we performed experiments in which each PI was added to replicating promastigote forms (5×10^7^ cells) at different concentrations and cell growth was monitored for 4 days in vitro. Only lopinavir and nelfinavir significantly reduced leishmanial development at 50 µM (*P*<0.05) (Fig. 1, HIV PIs). After this preliminary screen, each inhibitor was tested in the appropriate concentration range. All the PIs inhibited the parasite growth in a dose-dependent manner (Fig. 1). Nelfinavir and lopinavir induced a powerful reduction in the cellular growth rate from 20 and 25 µM concentration on, respectively. The IC_50_ after 48 hours was 15.12±1.1 µM and 16.47±0.8 µM, respectively. Amprenavir significantly impaired parasite multiplication only after 48 hours of growth at 100 µM (*P*<0.05). This drug at 250 µM virtually blocked parasite proliferation, while at 500 µM, parasite death was detected (Fig. 1, Amprenavir). The IC_50_ after 48 hours was 62.0±2.1 µM. Indinavir only at the highest concentration and after 72 hours of cultivation significantly altered parasite growth (Fig. 1, Indinavir). Conversely, saquinavir at the highest concentration used only marginally diminished the *L. amazonensis* proliferation (Fig. 1, Saquinavir). Pepstatin A was assayed in parallel for comparative purposes. This inhibitor powerfully diminished parasite growth at 50 µM (data not shown). DMSO did not significantly affect parasite growth behavior.

### Effect of HIV PIs on the ultrastructure

**Figure 2:**
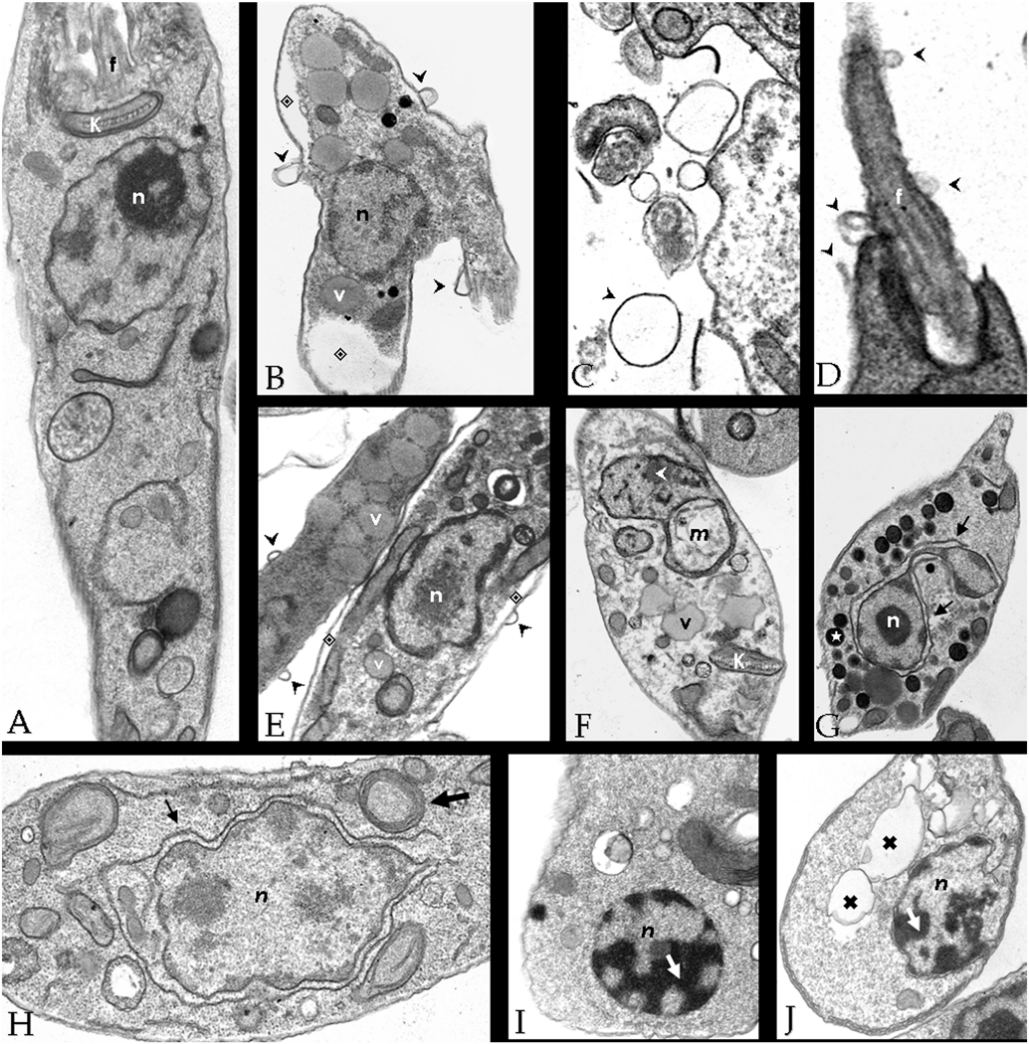
Ultrastructural changes observed in *L. amazonensis* after HIV PIs treatment. Parasites (1×10^8^ cells) from 48-hours cultures were inoculated in fresh medium in the absence (A) or in the presence of nelfinavir at 15.12 µM (IC_50_) (B–H) or lopinavir at 16.47 µM (IC_50_) (I–J) and incubated for 4 (B–E), 6 (F–G), 8 (H) and 24 (I–J) hours. Subsequently, cells were processed for transmission electron microscopy. An intense flagellar and plasma membrane shedding (black arrowheads) was seen after 4 hours of treatment with both inhibitors (B–D). Some effects were exclusive of nelfinavir, such as cytoplasm shrink (B and E, 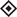), increase in the number of intracellular vesicles, resembling acidocalcisomes (G, ★) and lipid inclusions (E and F, v). Both drugs induced nuclear wrapping by the endoplasmic reticulum (G and H, black arrows), mithocondrial swelling (F, white arrowheads) and myelin-like structures (H, larger arrow). In lopinavir treated cells, blocks of condensed chromatin were observed close to the nuclear envelope (I, white arrow), as well as enlarged vesicles (J, 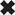). n - nucleus; k - kinetoplast; f - flagellum and m - mithocondrion. Some of the ultrastructural alterations described for nelfinavir (B–H) were also visualized with lopinavir, for simplicity, these phenomena were represented only by nelfinavir treated cells.

Based on the efficacy of the HIV aspartyl PIs in diminishing the growth of *L. amazonensis*, particularly nelfinavir and lopinavir, we next investigated the effect of these inhibitors in the *Leishmania* ultrastructure, by transmission electron microscopy. For this purpose, the morphology of non-treated cells (Fig. 2A) was compared with the ultrastructure of PIs-treated protozoa (Fig. 2B–J). Results showed blebs detaching from the entire cell surface (Fig. 2B–C, **arrowheads**), including the flagellar membrane (Fig. 2D, **arrowheads**), after 4 hours of treatment using the IC_50_ concentration (15.12 µM to nelfinavir and 16.47 µM to lopinavir). Interestingly cells treated with nelfinavir, but not with lopinavir, presented cytoplasm shrinking (Fig. 2B,E, 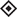), a higher number of vesicles, which according to their electron-density, probably corresponds to lipid-containing compartments (Fig. 2E,F, **v**) or acidocalciosomes (Fig. 2G, ★). Cells treated with both drugs presented the nucleus surrounded by endoplasmic reticulum (Fig. 2G–H, **black arrows**), mitochondrial swelling (Fig. 2F, **white arrowhead**) and myelin-like structures (Fig. 2H, **larger arrow**). After 24 hours of treatment with lopinavir, extensive blocks of condensed chromatin were observed close to the nuclear envelope (Fig. 2I–J, **white arrows**), which is suggestive of apoptosis, as well as enlarged vesicles (Fig. 2J, 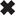). These effects were commonly visualized in the drug-treated population.

### Effect of HIV PIs on *Leishmania* aspartyl peptidase

**Figure 3:**
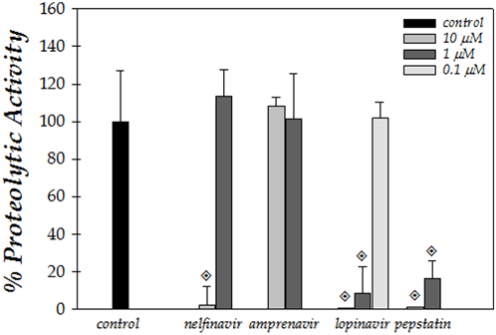
Effect of aspartyl PIs on *Leishmania* aspartyl peptidase activity. *L. amazonensis* soluble extract was incubated with 2 µM HIV-1 peptidase substrate for 15 min at 37°C in 100 mM sodium acetate, 1 M sodium chloride, 1 mM EDTA, 1 mM DTT, 10% DMSO, 1 mg/ml BSA, pH 4.7, in the absence (control) or in the presence of different concentrations of the following PIs: nelfinavir, amprenavir, lopinavir and pepstatin A (0.1 to 10 µM). The control (16.6±4.5 arbitrary fluorescence units) was taken as 100%. The values represent the mean±standard deviation of three independent experiments performed in triplicate. Symbol denotes systems treated with PIs that had a rate of substrate hydrolysis significantly different from the control (*P*<0.01; Student's t test).

The effect of distinct HIV aspartyl PIs on the hydrolytic activity of *L. amazonensis* extract over HIV-1 peptidase substrate was determined. To this end, nelfinavir, amprenavir and lopinavir, as well as pepstatin A were tested in concentrations ranging from 0.1 to 10 µM. The inhibitory capability of these compounds was very divergent (Fig. 3). Lopinavir was by far the most effective inhibitor, reducing the proteolytic hydrolysis of the substrate by approximately 90% at 1 µM, and virtually abolishing the proteolytic activity at 10 µM, while at 0.1 µM, the inhibitor exerted no significant effect in relation to control. Nelfinavir exhibited an inhibition of approximately 98% at 10 µM, however, at 1 µM, the inhibitor was ineffective, while amprenavir even at 10 µM did not significantly inhibited the hydrolysis of the substrate by *L. amazonensis* extract. The inhibition of hydrolysis by lopinavir was comparable to that exerted by pepstatin A, which is a prototypal inhibitor of aspartyl-type peptidases (Fig. 3).

### Effect of HIV PIs on the interaction of *Leishmania*-macrophage cells

**Figure 4:**
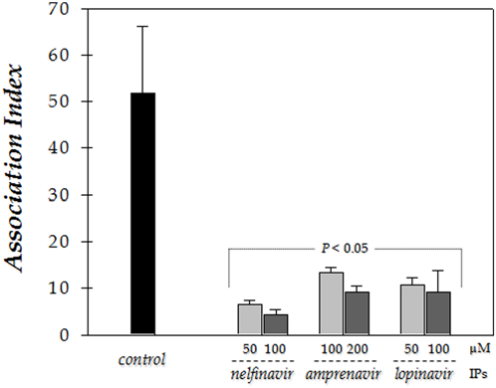
Effect of the pre-treatment of promastigotes with HIV PIs on the *Leishmania amazonensis*–macrophage interaction. The promastigotes of *L. amazonensis* were treated or not with nelfinavir, amprenavir or lopinavir (as indicated) for 1 hour prior to macrophage–parasite interactions, then the cells were washed with PBS. Parasites maintained their viability under this experimental condition (see Methods section). Macrophages were then infected with promastigote forms for 1 hour at 37°C, and macrophage monolayers were washed with PBS to remove unbound parasites. The association index was determined after 1 hour of infection by light microscopy, counting at least 200 cells in each of duplicated coverslips. Each bar represents the mean±standard error of at least three independent experiments.

**Figure 5:**
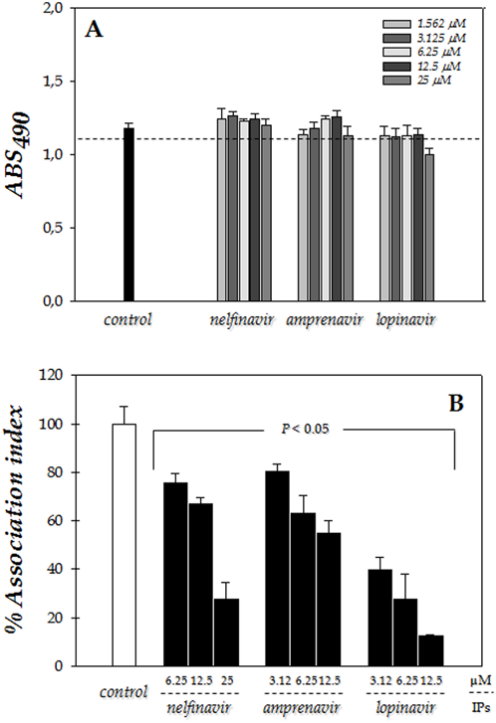
Toxicity of HIV PIs in mouse peritoneal macrophages and susceptibility of intracellular parasites to HIV PIs in macrophages. Initially, the macrophages (1×10^5^ cells) were incubated in a 96-well plate for 24 hours in the absence or in the presence of different concentrations (as indicated) of the following PIs: nelfinavir, amprenavir and lopinavir. After this period, the viability of the macrophage cells was determined spectrophotometrically at 490 nm by means of MTT assay. The dotted line separates the graphic in two portions: minor than and equal or major than 95% of macrophage viability. Control represents untreated macrophages (A). After that, we studied the susceptibility of intracellular parasites to HIV PIs in macrophages. Macrophages were infected with promastigotes of *L. amazonensis* for 1 hour at 37°C, followed by exhaustive washing in PBS and then the interaction was incubated for additional 24 hours in the absence (control), or in the presence of nelfinavir, amprenavir or lopinavir as indicated. The concentrations employed correspond to those in which more than 95% of the macrophage cells were viable as demonstrated in (A). Finally, the association index was determined by light microscopy, counting at least 200 cells in each of duplicated coverslips. Control system is shown as 100%. Each bar represents the mean±standard error of at least three independent experiments (B).

The effects of PIs on the *L. amazonensis*-macrophage interaction are shown in Fig. 4 and 5B. The promastigote forms of the parasite were treated for 1 hour before interaction with the PIs that showed more effectiveness in suppressing parasite proliferation in vitro, i.e., nelfinavir, lopinavir and amprenavir. At the drug concentrations tested (50 and 100 µM for nelfinavir and lopinavir; and 100 and 200 µM for amprenavir), the parasites maintained their viability after the treatment for 1 hour with each PI, as judged by their morphology, motility and propidium iodine staining, in which more than 95% of the promastigotes were viable (data not shown). Then, the drugs were washed away and the parasites were allowed to interact with macrophages for 1 hour, free parasites were removed and the cells incubated for an additional hour, then the association indexes were determined. We showed that nelfinavir was the most potent inhibitor of the interaction process between *L. amazonensis* and macrophage cells, showing a clear dose-dependent inhibition profile, where the inhibition increased from 86 to 93% (in relation to control) as nelfinavir concentration rose from 50 to 100 µM. When parasites were pretreated with 100 or 200 µM of amprenavir, the association indexes were, respectively, 75 and 81% lower as compared to the control system. Lopinavir was also capable of interfering in the interaction process; however, a dose-dependent inhibition profile was not prominent, being around 80% either at 50 or 100 µM (Fig. 4).

Given that PIs can interfere in the early steps of parasite infection, since the inhibitors were added exclusively to *Leishmania* promastigotes and that the interaction process was stopped with only 1 hour, we resolved to investigate the association index of *L. amazonensis* with macrophage cells during the in vitro treatment for 24 hours with aspartyl PIs used in the chemotherapy of HIV. First of all, we tested the effect of these three PIs alone on the mouse peritoneal macrophages viability by using the MTT assay. A significant deleterious effect was only observed with lopinavir at 25 µM, while amprenavir also at this concentration was in the boundary between non-toxic and toxic effects (Fig. 5A). Therefore, in the next section of these experiments, we used the PIs at the maximal concentrations in which more than 95% of macrophages were viable after 24 hours of treatment. Finally, the macrophages were preinfected with *L. amazonensis* for 1 hour, unbound parasites were washed away, and then treated with nelfinavir, lopinavir or amprenavir for 24 hours. Our results evidenced that all the tested aspartyl PIs notably reduced intracellular number of *Leishmania* in macrophages in a dose dependent manner.

### Effect of HIV PIs on the expression of classical leishmanial peptidases

**Figure 6:**
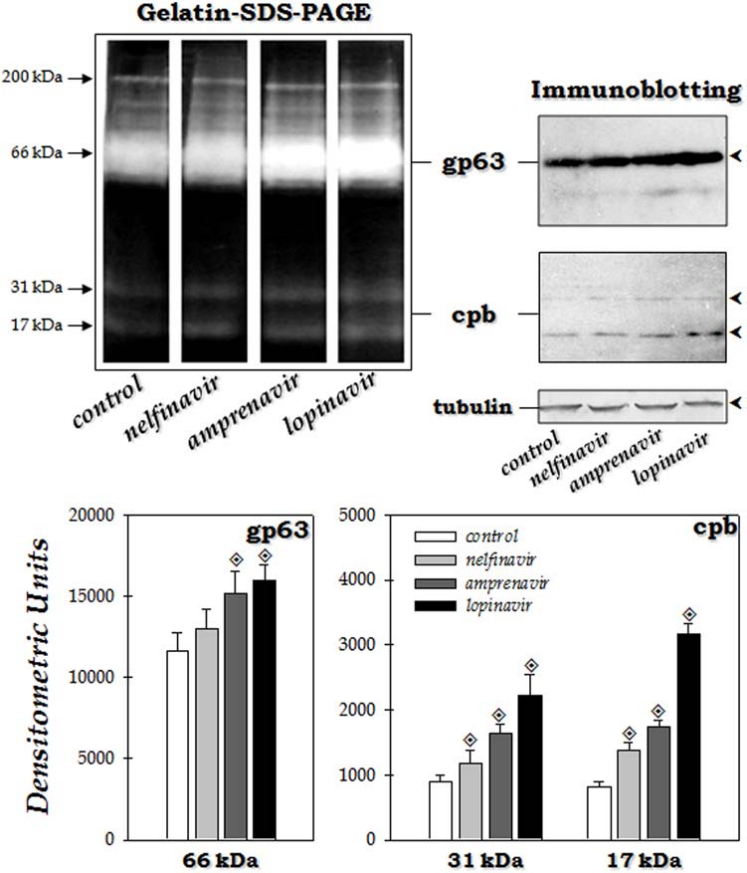
Effect of HIV PIs on peptidase expression by *L. amazonensis.* Differential peptidase expression observed in *L. amazonensis* promastigote forms grown in the absence (control) or in the presence of nelfinavir, amprenavir and lopianvir at 100 µM. Gelatin-SDS-PAGE: The gel strips, containing the equivalent to 5×10^6^ cells of parasite extract obtained after 4 hours of incubation with each inhibitor, were incubated at 37°C for 48 hours in 50 mM sodium phosphate buffer (pH 5.5) supplemented with 2 mM DTT. Numbers on the left indicate relative molecular mass of the peptidases, expressed in kilodaltons (kDa). Immunoblotting: anti-gp63 or anti-cpb antibodies were employed to detect gp63- and cpb-like molecules, respectively, in the whole cellular extract from *L. amazonensis* grown in the absence (control) or in the presence of nelfinavir, lopianvir and amprenavir for 4 hours at 26°C; Anti-tubulin monoclonal antibody was used as a control for sample loading in the gels. The graphics represent the densitometric measurements of the proteolytic halos observed in the gelatin-SDS-PAGE. The values represent mean±standard deviation of three independent measurements. Similar densitometric analyses were performed using the reactive polypeptides detected in the immunobloting assays (data not shown). Symbol denotes systems treated with PIs that had densitometric units significantly different from the control (*P*<0.05; Student's t test).

In this set of experiments we aimed to assess if the stress induced by the PIs would led to any change in the pattern of peptidase production by *L. amazonensis*. Cells were incubated for 4 hours in the presence of nelfinavir, lopinavir and amprenavir at 100 µM and assayed by zymography. At this experimental condition, the parasites maintained their viability, as judged by their morphology, motility and propidium iodine staining, in which more than 95% of the promastigotes were viable (data not shown). The proteolytic profile of *L. amazonensis* in gelatin-SDS-PAGE at pH 5.5 supplemented with DTT has been previously described and is composed mainly of metallo- and cysteine peptidases [16], [17], [22]. The comparison of the peptidase expression by gelatin-SDS-PAGE in parasites incubated or not for 4 hours with the PIs revealed that both the 63-kDa metallopeptidase and the low-molecular mass cysteine peptidases had their activity enhanced when parasites were subjected to PIs (Fig. 6). In order to further determine the influence of the PIs on classical peptidases from *L. amazonensis*, we have performed western blotting analysis of these molecules employing anti-gp63 or anti-cpb antibodies. In agreement with the gelatin-SDS-PAGE analysis, the gp63 molecule (a 63 kDa polypeptide) and the cpb (17 and 31 kDa) were enhanced when the parasites were incubated with nelfinavir, amprenavir and lopinavir. In all the methods employed, lopinavir promoted the most pronounced effect. For sample loading control, we have revealed the western blotting with anti-α-tubulin, which showed no difference between the experimental systems (Fig. 6).

## Discussion

Leishmaniasis is one of the most neglected tropical diseases [1], and the HIV/AIDS pandemic has changed the natural history of leishmaniasis [23]. The number of co-infections is increasing dramatically, especially due to increased overlap of both diseases [2]. Additionally, the real impact of *Leishmania*/HIV co-infection is probably underestimated on the global scale due to deficiencies in the surveillance systems. The fact that leishmaniasis is not included among the AIDS-defining diseases contributes to this scarcity of information [24]. HIV infection can increase the risk of developing leishmaniasis by 100–2000 times [2]. In addition, visceralization of *Leishmania* strains, such as *L. amazonensis*, that are classically restricted to cutaneous leishmaniasis has often been observed in patients with *Leishmania*–HIV co-infection [2]. HIV infection also reduces the likelihood of a therapeutic response, and greatly increases the probability of relapse [25]–[27]. At the same time, visceral leishmaniasis promotes the clinical progression of HIV disease and the development of AIDS-defining conditions. Both diseases exert a synergistic detrimental effect on the cellular immune response because they target similar immune cells [2].

Patients with HIV infection are surviving longer and with an improved quality of life as a result of the institution of HAART, regimens that usually consist of a PI combined with at least two other antiretroviral drugs [28], [29]. PIs bind competitively to the cleavage site within the HIV-1 aspartyl peptidase enzyme. They render the enzyme non-functional and lead to the release of immature, non-infectious viral particles [30]. Moreover, it has been demonstrated that PIs have advantageous effects on some opportunistic infections caused by fungal pathogens, and also some protozoa, both known to be the major causes of morbidity and mortality in AIDS subjects, due to restoration of the host immune system and a direct action on the pathogens. Although there are several studies concerning *Leishmania*/HIV co-infection, some particularities of its epidemiology, pathogenesis, prophylaxis and especially of its treatment remain unclear and undefined [1], [31]. Based on these preceding evidences, we studied the implication of distinct HIV PIs on the human protozoan parasite *L. amazonensis*, focusing on the effects in the peptidase activity, parasite proliferation, ultrastructure and interaction with animal cells.

Promastigote forms of *L. amazonensis* were cultured with pepstatin A, nelfinavir, indinavir, saquinavir, lopinavir and amprenavir up to 96 hours, and the parasite survival was analyzed. Pepstatin A promoted a powerful reduction on the growth of *L. amazonensis*. Pepstatin-like drugs, however, are not used clinically because of their metabolism in the liver and rapid clearance from blood [32]. Nelfinavir and lopinavir robustly inhibited the *L. amazonensis* growth at 25 µM, in contrast to the other PIs tested. It should be pointed out that the inhibitory effect of HIV PIs in vitro were observed at substantial PI concentrations (µM range), much higher than those needed for HIV peptidase inhibition (on the nM order) [33]. This probably reflects a much lower affinity of these drugs for a yet unidentified target in *Leishmania* compared with their high affinity for HIV peptidase [33].

Parasitic protozoa of the genus *Leishmania* are a biologically diverse group of microorganisms. Taxonomic studies of field *Leishmania* isolates indicate a tremendous diversity within this genus [34], and genetic diversity may have a direct impact on relevant properties of the organisms, including distinct clinical manifestations and drug susceptibility [35]. So, it is not surprising that the effect of HIV PIs on *Leishmania* species responsible for visceral or mucocutaneous leishmaniasis seems to be very distinct. Savoia and colleagues [14] reported the IC_50_ for *L. major* promastigote in the 10 µM range for indinavir and saquinavir after 24 hours of growth, while *L. infantum*, responsible for visceral leishmaniasis, was inhibited only partially (about 30%) at the highest concentration tested (50 µM) and only after 72 hours of growth [14]. In the course of this manuscript preparation, Trudel and colleagues [36] reported the effects of nelfinavir, ritonavir and saquinavir on the extracellular growth of *L. infantum* promastigotes. No significant alteration in parasite growth was reported in drug concentrations up to 25 µM, after 72 hours of incubation [36]. Our results showed an IC_50_ for *L. amazonensis* in the 15 µM range for nelfinavir or lopinavir after 24 hours of growth.

The effectiveness of HIV PIs in treating parasitic infections may be associated to their capacity to modulate or block the cell proteasome or to promote apoptosis [9]. Alternatively, it could act directly on aspartyl peptidases produced by protozoa. This proteolytic class has already been described in *L. amazonensis* [37] and *L. mexicana* [38]. In the latter, it was also shown that diazo-acetyl-norleucinemethylester (DAN), an aspartyl peptidase inhibitor, significantly reduces promastigote growth. Moreover, in the present report, we demonstrated that nelfinavir and lopinavir powerfully inhibited the hydrolysis of HIV-1 peptidase substrate, at 10 and 1 µM, respectively. Although it seems reasonable to assume that the HIV PIs target *Leishmania* aspartyl peptidase and/or proteasome, the possibility of unrelated effects of inhibitors on *L. amazonensis* should also be considered, such as nonspecific or generally toxic effects on parasite cells. In this context, electron-micrographic examination of protozoan cells exposed to nelfinavir or lopinavir revealed some peculiar alterations in vital cellular structures, such as cytoplasmic membrane and internal cellular structures, suggesting irreversible metabolic injuries that culminate in the parasite cell death.

Some of the ultrastructural alterations observed in *L. amazonensis*, such as increase in the number of vesicles and wrapping of the nucleus by the endoplasmic reticulum, are suggestive of autophagy. In this sense, recent data have provided evidence for the existence of different forms of programmed cell death. In addition to the long time established existence of apoptosis, autophagy has been increasingly described in eukaryotic cells [39]. Autophagy is a normal degradative process that exists in all eukaryotic cells and is stimulated in response to a variety of environmental stresses, which necessitate the use of autophagic mechanisms to enable cellular survival [40], [41]. Autophagy involves the sequestration of cytosol or cytoplasmic organelles within double membranes, thus creating autophagosomes (also called autophagic vacuoles). Autophagosomes subsequently fuse with endosomes and eventually with lysosomes, thereby creating autophagolysosomes or autolysosomes. In the lumen of these latter structures, lysosomal enzymes operating at low pH then catabolize the autophagic material [42], [43]. Low levels of autophagy are considered to be responsible for better survival, while abnormally high levels of autophagy might be responsible for cell death [43]. Up to now, the deregulation events responsible for uncontrolled autophagic activity have not been well characterized [44]. Additionally, in contrast to mammalian cells, the autophagic process in unicellular organisms is less understood. Finally, the accumulation of autophagic vacuoles can precede apoptotic cell death [44]. Indeed, *Leishmania* cells grown in the presence of lopinavir for 24 hours displayed a chromatin condensation, which is one of the hallmarks of apoptosis. Therefore, it would be interesting to further explore the mechanism of cell death induced by HIV PIs in *Leishmania*.

In the opportunistic fungal pathogen *Cryptococcus neoformans*, indinavir selectively inhibits the production of some virulence factors, such as urease and peptidase. Additionally, this inhibitor also interfered with polysaccharide capsule formation, the major virulence factor produced by this microorganism [45]. In this context, we aimed to assess if the PIs would induce any change in the peptidase expression by *L. amazonensis*. Over the past few years it has become clear that peptidases from the pathogenic trypanosomatids play an important role in several steps of the host infection: adhesion, penetration, intracellular survival, replication, differentiation, infectivity, immune evasion and nutrition. Trypanosomatids present a large and varied array of intracellular and/or extracellular peptidases whose regulated expression entails specific functions in the parasite life-cycle stages [18]. *Leishmania* spp. contain multiple, highly active intracellular cysteine peptidase activities and an abundant intracellular or surface-associated metallopeptidase, named gp63 or leishmanolysin [16]-[18]. The majority of the studies so far developed have dealt with only three types of cysteine peptidases, designated CPA, CPB and CPC [16]. The generation of null mutants for CPA, CPB and CPC genes in *L. mexicana* has provided the first genetic support for the key role of leishmanial cysteine peptidases in parasite virulence, and hence their validation as drug targets [16]. Accordingly, it has been demonstrated that cysteine peptidases are preferentially expressed in virulent, as opposed to avirulent *L. amazonensis* promastigotes [22]. Leishmanolysin is also involved in *Leishmania* virulence and pathogenicity. The expression of the enzyme is increased in metacyclic promastigotes and it may be a ligand involved in the interaction of the parasite with defensive systems of the host, including components of the complement system and the macrophage surface. This enzyme can also play a role in intracellular amastigote survival within macrophage phagolysosomes. Nevertheless, data from gp63 knockouts are conflicting [17].

In this context, contrary to the view that HIV PIs would lead to a reduction in peptidase levels, as described for virulence factors in fungi, our data revealed a significant increase in the expression of these molecules when parasites were subjected to PIs before protein extraction. Curiously, a similar effect was described when *L. amazonensis* was treated with parthenolide, which is a sesquiterpene lactone purified from the hydroalcoholic extract of aerial parts of *Tanacetum parthenium* [46]. One hypothesis could be that the HIV PIs are inhibiting an aspartyl peptidase that should be otherwise degrading some of the gp63 and CPB peptidases. Accordingly, the degradation of the HIV-1 peptidase substrate was substantially inhibited by HIV PIs. An alternative hypothesis could be that the HIV PIs are exerting stress, or some other non-specific effect, on the promastigotes that leads to changes in parasite gene expression. Indeed, stresses such as heat shock are important to the differentiation of *L. amazonensis* into axenic amastigotes, whereupon cysteine peptidase gene expression, and hence activity, is upregulated [37], [47].

The murine infection is one of the best characterized experimental models for studying *Leishmania*-mammalian host cells interplays [48]. Data from infection of peritoneal mouse macrophages by *L. amazonensis* revealed that the parasites pretreated with PIs had association indexes considerably lower in comparison to control system. Taken together, these results suggest interference in early steps of macrophage infection either through modulation of yet unidentified virulence factors, blockade of ligand/receptor binding, suppression of ligand/receptor expression or due to direct non-specific toxic effects of the inhibitor. It should be pointed out that these interaction inhibition profiles were not caused by a decreased viability of *L. amazonensis* cells, as assessed by propidium iodide staining. The identification of the molecular mechanism by which the HIV PIs interfere with promastigote interaction with mammalian cells remains an open question.

It has been previously demonstrated that inhibitor compounds effectiveness may depend on the developmental stage of the parasite. For instance, *L. mexicana* amastigotes developing within macrophages during human infection are more sensitive to lactacystin than promastigotes developing in culture medium [49], which may be explained by a distinct action of the drugs on promastigotes alone and on amastigotes in an intracellular environment. In order to assess if the same applies to HIV PIs, we evaluated the effect of nelfinavir, amprenavir and lopinavir on the *Leishmania* development inside target cells. The drug concentrations employed in this assay showed no direct toxicity on macrophages, while they drastically reduced the association index at considerably lower doses than those required to interfere with promastigotes. Whether the inhibitors are acting directly in the amastigotes or indirectly, modulating the killing capability of the macrophages, remains an open question. Alternatively, macrophages could concentrate higher levels of PIs. Accordingly, it was shown in *L. chagasi* that higher drug concentrations are necessary to interfere with axenic amastigotes when compared to intracellular amastigotes [36]. Finally, even the lower doses required to inhibit amastigote development are still higher than those needed for the inhibition of HIV progression in humans. However, we have to consider that HAART consists of a combination of antiretroviral drugs and the pharmacodynamics of an in vitro model is very different from those in humans. Moreover, the HIV PIs were designed to fit viral peptidases and may thus have a lower affinity for aspartyl peptidases of *L. amazonensis*. Additional in vivo studies will help to elucidate this issue, and also further efforts should be directed to find new PIs that are more specific for these parasite enzymes.

In Brazil, the clinical forms found among coinfected cases are 43% mucocutaneous, 37% visceral, and 20% cutaneous leishmaniasis [50], a clinical pattern that differs from that found in southern Europe, where typical visceral leishmaniasis represents 88% of coinfected cases; 8% of cases are atypical because of intestinal, lung, or other parasite colonizations, about 5% are cutaneous, and 0.3% are combined cases of visceral and cutaneous leishmaniasis [2], [51]. In the face of leishmaniasis distribution in Brazil, its spread to important cities and the overlap with HIV-infected individuals, it is critical to further comprehend the sophisticated interplays among *Leishmania*, HIV and macrophages. In addition, there are many unresolved questions related to the management of *Leishmania*-HIV-coinfected patients. For instance, the efficacy of therapy aimed at controlling each pathogen in coinfected individuals remains largely undefined. The results presented herein add new in vitro insight into the wide spectrum efficacy of HIV PIs and suggest more detailed studies about the synergistic effects of classical antileishmanial compounds and HIV PIs in macrophages coinfected with *Leishmania* and HIV-1, especially in the light that sodium stibogluconate can stimulate HIV-1 replication in vitro [52], and that *Leishmania* infection enhances HIV-1 replication [53], [54]. Macrophages infected with HIV-1 allow the intracellular growth of an otherwise non-pathogenic protozoan, *Blastocrithidia culicis*, which illustrates the susceptibility of these cells to parasite infections [55]. Finally, it was recently reported that *L. infantum* promastigotes enhance HIV-1 replication in monocyte-derived macrophages at late time points in the virus growth curve but also, surprisingly, a reduction in HIV-1 production is seen during the initial days after infection. This early effect is caused by a *Leishmania*-mediated inhibition of virus entry into monocyte-derived macrophages through the action of lipophosphoglycan (LPG), the major promastigote surface glycolipid. Altogether, these data suggest the establishment of complex interactions in *Leishmania*-HIV common natural host cells [56].
